# How Many Clocks, How Many Times? On the Sensory Basis and Computational Challenges of Circadian Systems

**DOI:** 10.3389/fnbeh.2018.00211

**Published:** 2018-09-11

**Authors:** Jason Somers, Ross E. F. Harper, Joerg T. Albert

**Affiliations:** ^1^Ear Institute, University College London London, United Kingdom; ^2^The Francis Crick Institute London, United Kingdom; ^3^Centre for Mathematics and Physics in the Life Sciences and Experimental Biology (CoMPLEX), University College London London, United Kingdom; ^4^Department of Cell and Developmental Biology, University College London London, United Kingdom

**Keywords:** circadian clock, biological oscillator, multisensory integration, bayesian modeling, *Drosophila melanogaster*, sensory conflict

## Abstract

A vital task for every organism is not only to decide *what* to do but also *when* to do it. For this reason, “circadian clocks” have evolved in virtually all forms of life. Conceptually, circadian clocks can be divided into two functional domains; an autonomous oscillator creates a ~24 h self-sustained rhythm and sensory machinery interprets external information to alter the phase of the autonomous oscillation. It is through this simple design that variations in external stimuli (for example, daylight) can alter our sense of time. However, the clock’s simplicity ends with its basic concept. In metazoan animals, multiple external and internal stimuli, from light to temperature and even metabolism have been shown to affect clock time. This raises the fundamental question of cue integration: how are the many, and potentially conflicting, sources of information combined to sense a single time of day? Moreover, individual stimuli, are often detected through various sensory pathways. Some sensory cells, such as insect chordotonal neurons, provide the clock with both temperature and mechanical information. Adding confusion to complexity, there seems to be not only one central clock in the animal’s brain but numerous additional clocks in the body’s periphery. It is currently not clear how (or if) these “peripheral clocks” are synchronized to their central counterparts or if both clocks “tick” independently from one another. In this review article, we would like to leave the comfort zones of conceptual simplicity and assume a more holistic perspective of circadian clock function. Focusing on recent results from *Drosophila melanogaster* we will discuss some of the sensory, and computational, challenges organisms face when keeping track of time.

## Introduction

A good decision is not absolute. It varies depending on context. Foraging for food can be a good or bad decision depending on the presence of a looming predator. The key is to optimize behavior for the current context. But what if the context is in perpetual flux? Owing to the spin of our planet on its longitudinal axis, the vast majority of life on earth exists in 24-h cycles of environmental change. The sun rises and sets. Dawn comes and goes. Temperatures change, and both predator and prey alike sleep and wake. Thus, good decisions depend as much on *when* to do, as they do on *what* to do.

This critical importance of time for decision-making necessitates the existence of internal clocks. These “circadian” oscillators give temporal structure to behavior. The circadian system of *Drosophila melanogaster* presents a remarkable tool to investigate how external environmental changes can impact internal timekeeping that best prepares an organism for time-appropriate tasks. The circadian clock has a set period (i.e., one full cycles takes ~24 h) and its time, or phase, can be adjusted by incoming sensory information. This flexibility is paramount to a system that is orchestrating numerous behavioral and physiological processes while external stimuli are constantly changing.

In this review article we set out to highlight the complexities of the *Drosophila* *melanogaster* circadian clock, in which “decisions” must be made with regard to external environmental cues. These fundamental timing decisions are made by individual neurons in the context of multimodal sensory information. By discussing the complexities that exist on the molecular, cellular, network and behavioral level, we propose computational approaches that may be fruitful to gain further insight into the nuances of circadian systems.

## The Molecular Clock

**Figure 1:**
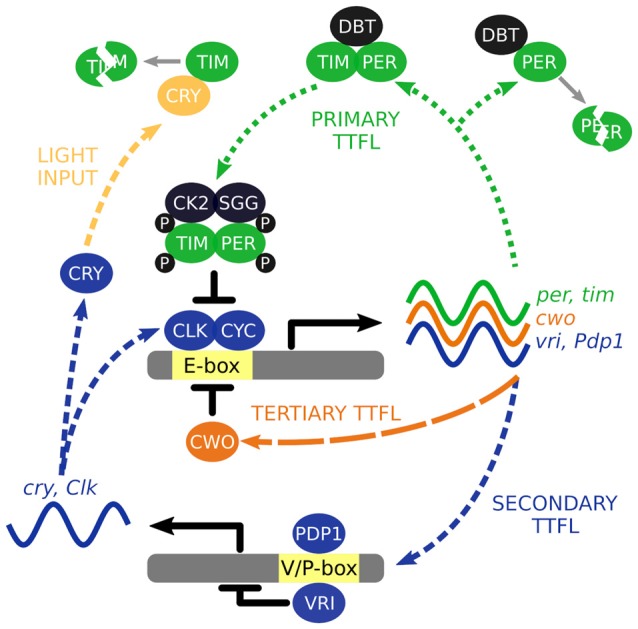
The interlocked core transcription/translation feedback loops (TTFL) of *Drosophila* *melanogaster*. The primary TTFL is initiated by the CLK-CYC mediated transcription of the *per* and *tim* genes. The PER protein is inherently unstable and DBT will direct it for degradation unless PER is stabilized by TIM. PER and TIM are phosphorylated (P) by CK2 and SGG to negatively regulate their own expression by repressing CLK-CYC activity. VRI and PDP1 are also produced by CLK-CYC activity, each of which have potentially opposing effects and offset timing to modulate *Clk* and *cry* transcription. Light activation of CRY leads to the degradation of TIM acting as a molecular reset of the TTFL cycling. CWO is a part of third TTFL that also acts as a repressor of CLK-CYC activity.

To understand how the wider circadian system keeps time, we must first begin with the oscillatory building blocks that comprise it. Time is computed at the level of individual cells and integral to this cellular timekeeping is a molecular clock that is driven by the autonomous oscillations of so called “clock genes”. The autonomous oscillators are driven by a series of molecular transcriptional/translational feedback loops (TTFL) of which components autoregulate their own expression (Figure 1), reviewed in Hardin (2011).

The molecular clock starts with the transcription factors, CLOCK (CLK) and CYCLE (CYC), activating transcription of the primary TTFL genes, *per* and *tim*, through binding to E-box regions in their gene promoters (Hao et al., 1997; Allada et al., 1998; Darlington et al., 1998; Rutila et al., 1998). Transcription occurs between ~ZT4 to ~ZT18 (where ZT0 is lights on and ZT12 is lights off) and translation of these mRNAs generate the protein products PERIOD (PER) and TIMELESS (TIM). The PER protein is inherently unstable, and is rapidly targeted for proteasomal degradation by the kinase DOUBLETIME (DBT; Kloss et al., 1998, 2001; Price et al., 1998). However, the PER-DBT dimer can be stabilized through dimerization with TIM allowing the PER-TIM-DBT complex to accumulate in the cytosol, around 6–8 h after *per* and *tim* transcription activation or ~ZT12 (Curtin et al., 1995; Gekakis et al., 1995; Price et al., 1995; Zeng et al., 1996).

Phosphorylation of PER and TIM by the kinases, CK2 and SGG (Martinek et al., 2001; Lin et al., 2002; Akten et al., 2003), initiates nuclear accumulation of PER and TIM (Nawathean and Rosbash, 2004; Top et al., 2016). Inside the nucleus, the active form of PER inhibits CLK-CYC activation of *per* and *tim* transcription between ~ZT18 to ~ZT4 (Lee et al., 1998, 1999; Bae et al., 2000; Menet et al., 2010). Consequently, PER and TIM concentrations decline in the cytosol, reducing nuclear abundance of PER and TIM, and ultimately removing the inhibition of CLK-CYC activity. The molecular cycle then starts again, *circa* 24 h later.

The primary TTFL is bolstered by additional interlocked loops. In a second TTFL CLK-CYC also activates transcription of *vrille* and *Pdp1ɛ/δ* between ~ZT4 and ZT16 (Blau and Young, 1999; Cyran et al., 2003). VRILLE (VRI) protein concentration peaks several hours before PDP1ɛ/δ (~ZT14 vs. ~ZT18) and both act at VRI/PDP1-boxes to modulate *Clk* and *cry* transcription (Cyran et al., 2003; Glossop et al., 2003). PER-TIM-DBT activity from the primary TTFL inhibits CLK-CYC activity interlinking the two loops. The *cry* gene encodes a blue-light photoreceptor that upon photic activation promotes TIM degradation essentially acting as a molecular reset switch of the primary TTFL (Emery et al., 1998; Stanewsky et al., 1998). In the third TTFL, CLK-CYC promotes expression of *clockwork orange* that competitively binds to the same E-box regions to negatively regulate CLK-CYC mediated transcription (Kadener et al., 2007; Lim et al., 2007; Matsumoto et al., 2007).

## The Central Clock—A Network of Coupled Oscillators

**Figure 2:**
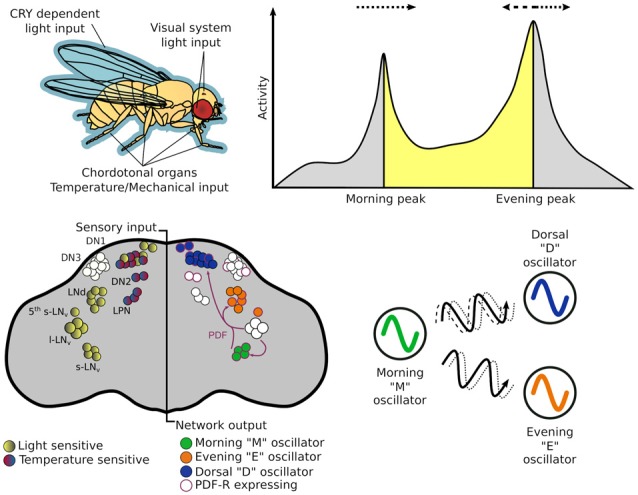
The circadian system of *Drosophila* *melanogaster* from input stimulus to output behavior. Light information may enter the circadian system through the cuticle of the fly to directly act on CRY, which is expressed in many central and peripheral clock cells. Alternatively, it may enter via sensory cells of the visual systems—compound eye, H-B eyelets or the ocelli that make direct—or indirect — synaptic contact with central clock neurons. While flies have many thermo - and mechano-sensing organs, chordotonal organs (ChOs) have been directly implicated in communicating both temperature and mechanical stimuli to the circadian system. The neuronal populations of central pacemakers differ in their sensitivity to external stimuli as well as their neurochemical and electrical connectivity. Pacemaker neurons expressing CRY preferentially entrain to light cues while others not expressing CRY have demonstrated preference for temperature cues. Neurochemical differences can denote distinct functional groups—for example the M oscillator neurons express and release pigment dispersing factor (PDF) that can lead to phase changes in molecular oscillations of cells expressing PDF-R. This can result in phase advances or delays of downstream processes that in turn can advance or delay behavioral outputs.

If the oscillating proteins of the TTFLs are the gears that direct timekeeping, the central pacemaker neurons are analogous to a synchronizer that sets a coherent time in context with the external environment. The architecture of this central pacemaker in flies consist of ~150 neurons comprising distinct subgroups identified in the brain through cytological staining of clock genes (Figure 2; Helfrich-Förster and Homberg, 1993; Helfrich-Förster, 1995). The subgroups are named after their morphology and anatomical location; each brain hemisphere has four small ventral lateral neurons (s-LN_v_) that express the pigment dispersing factor (PDF), a fifth PDF-negative s-LN_v_, four large PDF-positive ventral lateral neurons (l-LN_v_), six dorsal lateral neurons (LN_d_), 17 group one dorsal neurons (DN1), two group two dorsal neurons (DN2), ~40 group three dorsal neurons (DN3) and three lateral posterior neurons (LPN; Helfrich-Förster et al., 2007; Nitabach and Taghert, 2008; Schubert et al., 2018).

The dual oscillator model, originally hypothesized for nocturnal rodents (Pittendrigh and Daan, 1976), has historically been used to also describe the *Drosophila* crepuscular activity patterns, proposing separate autonomous oscillators that control activity peaks observed at dusk and dawn. The PDF+ LN_v_ are required for the fly’s morning activity peak (and free-running rhythms; Renn et al., 1999; Grima et al., 2004; Stoleru et al., 2004), while the LN_d_ and 5th PDF- LN_v_ are important for the evening peak (Grima et al., 2004), thus, these two groups are labeled the morning (M) and evening (E) oscillators respectively. In practice these designations are too strict as the roles and hierarchy of some cellular oscillators change under different environmental conditions (Rieger et al., 2006, 2009; Murad et al., 2007; Picot et al., 2007).

Current pacemaker models depict a variable—both neurochemically and electrically—coupled neuronal network that differentially responds to incoming multimodal stimuli (Yao and Shafer, 2014; Schlichting et al., 2016; Yao et al., 2016) reviewed in detail in Hermann-Luibl and Helfrich-Förster (2015); Top and Young (2018).

## Peripheral Clocks

In addition to the central pacemaker clocks, circadian oscillators also exist in several tissues outside the central nervous system and are likely to regulate organ or tissue specific functions. Peripheral clocks have largely been characterized through clock TTFL protein rhythms identified either by immunohistochemistry or by luciferase reporter expression being driven by clock gene promoters. Located in a number of tissues, peripheral clocks are heterogeneous in both their molecular machinery and their relationship to the central clock (Ito and Tomioka, 2016).

At the molecular level, CRY appears to act not only as a photoreceptor but also as a core component of the primary TTFL. In some peripheral clocks of *cry* mutants—in which CRY normally fulfils both roles—light entrainment in lost, however they also lose molecular oscillations of *per* and *tim* (Ivanchenko et al., 2001; Krishnan et al., 2001). In these peripheral clocks it appears CRY not only integrates photic information into the clock, but it also plays a role in driving oscillations of the clock, possibly acting as a repressor (Collins et al., 2006). Alternatively, CRY may act exclusively as a photoreceptor with persistent free running rhythms observed in certain tissues of *cry* mutants. This indicates that in these peripheral clocks CRY is not required to drive oscillations of the clock (Ito et al., 2008).

Phase relations between central clocks and peripheral clocks are also heterogeneous. For example, peripheral clocks in the Malpighian tubules and chemosensory sensilla can directly entrain to external stimuli and maintain entrained rhythms even without information from central clock neurons (Hege et al., 1997; Krishnan et al., 1999). A different phase relationship is observed between oenocytes and the central clock. PDF release from clock neurons into the hemeolymph can set the phase of the distally located oenocytes, which control rhythmic release of mating pheromones (Krupp et al., 2013). The different phase relationships identified between central and peripheral clocks add yet another layer of complexity to the timekeeping system.

## Locomotor Activity Rhythms

Circadian rhythms exist at the cellular and network levels, yet it is the appropriate timing of behaviors that makes this system adaptive for the organism. Behavioral output of a circadian clock is typically measured as the organism’s activity pattern; this is no different for *Drosophila*. Simple infra-red beam-breaking monitors record the activity of individual flies isolated in small glass tubes with enough food to last the duration of the experiment. These monitors are placed inside incubators in which the environmental conditions can be accurately controlled according to the test paradigm. In the simplest experiments, transitions between environmental conditions, or Zeitgebers (“time giver” in German), occur almost instantaneously (e.g., 12 h of light followed by 12 h of dark—LD). Here, *Drosophila* exhibits the previously mentioned bimodal activity pattern, with M and E activity peaks occurring at the Zeitgeber transition points. If the flies are then released into constant, free-running conditions, devoid of temporal information, rhythmic activity persists. However, the rhythms become almost exclusively unimodal possibly due to the merging of the previous M and E activity peaks (Wheeler et al., 1993; Helfrich-Förster, 2000). The timing of this free-running activity peak is a common read-out of “clock time” or circadian time. By adjusting the timing of cue onset/offset, the entrainability of the autonomous oscillator, which adjusts subsequent free-running rhythms to new schedules, can be tested. The speed of entrainment and relative amplitudes of autonomous oscillations depend on the strength and mode (e.g., light, temperature, vibration) of the specific Zeitgeber signal. Indeed, the precise activity profile observed depends on both the environmental conditions and the genetic background of the fly (Schlichting and Helfrich-Förster, 2015).

## Entrainment

Photic information is communicated to clock neurons both directly via CRY, and indirectly via visual photoreceptors residing in the compound eyes, ocelli and Hofbauer-Buchner eyelets. CRY mediates cell-autonomous perception of light in a number of tissues that harbor circadian rhythms via its interaction with TIM in the primary TTFL (Plautz et al., 1997; Emery et al., 2000; Lin et al., 2001). This direct input pathway makes the *Drosophila* circadian system particularly sensitive to light, capable of entraining to low light intensities (0.03 lux; Bachleitner et al., 2007), and can exhibit significant phase shifts following brief light pulses (Levine et al., 1994; Egan et al., 1999; Vinayak et al., 2013). Visual pathways to the clock are not as well defined and do not reset the clock with the same efficiency as CRY dependent pathways (Emery et al., 2000; Helfrich-Förster et al., 2001). However, it appears that a subset of rhodopsin photoreceptors communicate visual photic information to the clock by a novel phototransduction pathway (Stanewsky et al., 1998; Ogueta et al., 2018).

Temperature cycles (TC) can also entrain the circadian clock, and although this can occur in a tissue autonomous fashion in peripheral clocks, signaling from peripheral sensors play an important role in entraining the central clock (Glaser and Stanewsky, 2005; Sehadova et al., 2009). Thermoreceptors, expressed in chordotonal organs (ChOs) have been identified in the thermotransduction pathway, relaying information to the clock across different TC regimes (Wolfgang et al., 2013; Chen et al., 2015). Furthermore, genes important to the structural integrity of ChOs have also shown importance for entrainment to TC (Glaser and Stanewsky, 2005; Sehadova et al., 2009). Specifically, *nocte* (*no circadian temperature entrainment*) was identified in a screen for mutants that could entrain to LD but not to TC (Glaser and Stanewsky, 2005). Interestingly, the *nocte* mutant cannot entrain to combined LD and TC, hinting at a role in a sensory integration pathway that appears to eventuate in the DN1 central clock neurons (Chen et al., 2018).

ChOs also play a role in entrainment to mechanical inputs and this appears dependent on where the ChO is located. ChOs are stretch sensitive organs that populate almost every exoskeletal joint mediating proprioception in the legs as well as hearing in the antennae (Kavlie and Albert, 2013). Flies with no functional ChOs fail to entrain to 12-h vibration/12-h silence cycles, whereas flies lacking only antennal ChOs entrain better than their wild type controls (Simoni et al., 2014). This may be another feedback loop in the circadian system where output activity feeds information back to the clock.

## Natural Conditions

In order to perform more ecologically relevant experiments, environmental conditions can also be shifted in relation to each other in order to closely mimic natural conditions. This can be taken even further by placing the activity monitors outside the predictable conditions of the laboratory in the real-world environment. Under semi-natural “Summer” conditions an extra activity peak is observed that coincides with the temperature maxima termed the afternoon (A) peak (Vanin et al., 2012). Evidence suggests that A peak activity is induced by activation of the TRPA1 thermosensor in the AC neurons, rather than the circadian clock pacemakers (Tang et al., 2013; Green et al., 2015). The ecological relevance of the A peak is hypothesized to be an escape response from afternoon heat which is made evident by the occurrence of this peak being largely environmentally controlled. Closer observations of the fly activity support this hypothesis. Inactive flies at a preferable temperature (28°C) quickly retreat to the relative coolness of their food at an uncomfortable temperature (31°C) and are subsequently induced to erratic hyperactivity at noxious temperatures (35°C; Menegazzi et al., 2012). The phase of the A peak, however, still appears to be modulated by both the clock and environmental conditions (Menegazzi et al., 2012; Vanin et al., 2012). Interestingly, a functional clock appears to suppress the A peak at non-noxious temperatures. Clock mutant flies, deficient in either PER or CLK, exhibit A peak activity under milder TC in which wild-type flies exhibit bimodal activity patterns (Currie et al., 2009; Menegazzi et al., 2012; Vanin et al., 2012). This could be the clock mutant’s lack of time perception being unaware of the daily afternoon peaks of temperature, whereas wild-type flies anticipate the regular afternoon increase in temperature knowing it is nothing to be concerned about until a critical threshold is crossed at noxious temperatures. This is a more explicit decision-making process governed by the circadian clock—knowing what stimuli to respond to and what stimuli can safely be ignored.

## Conflict Conditions

The complexity of the number of potential interactions that could occur between the central clock pacemakers and the peripheral clocks is only now starting to be realized. How many clocks contribute to the animal’s overall sense of time? The historical view that one master pacemaker clock actively sets the phase of all the downstream clocks is being challenged. Perhaps the central pacemaker sets the time of central processes and regional peripheral clocks set time locally? To test this, offset environmental conditions have been used to understand the molecular, and subsequent behavioral repercussions, of receiving potentially conflicting environmental cues. During antiphasic conflict of light and temperature (a 12-h maximal misalignment between the two cues), the activity patterns of the flies demonstrate preferential entrainment to light (Yoshii et al., 2010; Harper et al., 2016). An assessment of the relative strength of photic and thermic input under these conditions suggests light to be the victor. However, observation of fly activity under other misalignment schedules reveals that flies appear to follow temperature cues when the misalignment is short (2–4 h) and light cues when the misalignment is long (>7 h). Misalignment between this (5–7 h) produces a novel behavior of sustained activity over the course of misalignment (termed “plateau” or P behavior; Harper et al., 2016). P behavior was not observed in the clock-less *per* mutants or the light-input pathway impaired *cry* mutants. The *per* mutant generally displayed arrhythmic behavior with brief startle responses to environmental changes while, due to their lack of the CRY photoreceptor, *cry* mutants entrained preferentially to temperature cues. The lack of the P behavior in these two clock mutants during sensory conflict demonstrates that the behavior depends on a functional clock and is not the result of environmental masking. Furthermore, a severe dampening of PER oscillations in the central pacemakers under conflict conditions support the hypothesis that sensory conflict is causing the abnormal P behavior. This contrasts with results from a subsequent study in peripheral clocks. During similar conflict conditions, bioluminescent PER reporters expressed in peripheral clocks, revealed peripheral molecular rhythms remained entrained to the light cue (Harper et al., 2017). Again, CRY expression appears to be key for this light cue entrainment in these tissues as peripheral molecular rhythms in *cry* mutants follow the temperature cue under conflict conditions. The fact that molecular rhythms in these peripheral clocks do not collapse as observed in the central clock could indicate that these peripheral clocks do not contribute to locomotor rhythms or alternatively that asynchrony between central and peripheral clocks results in abnormal behavior. Further studies are required to unpack this relationship.

## Probabilistic Considerations and Future Computational Approaches

The “circadian clock” is a complex, highly interconnected system, which attempts to determine—or predict—the state of the world from noisy or incomplete data. Probabilistic modeling provides a powerful conceptual framework for the theoretical, and experimental, analysis of such a system.

In a circadian setting, we can think of Zeitgebers as observable variables, from which the clock must compute the otherwise unobservable time of day. Bayesian integration provides an optimal algorithmic method by which to combine different sources of information. Here, the goal of a Bayesian observer (e.g., the “circadian clock”) is to compute the conditional density function that specifies the probability of the time of day from the given external cues (e.g., light and temperature). Further complexity is added by the temporal structure of circadian data, requiring that probability distributions be calculated over sequences of observations. Hidden markov models (HMMs) are one way of representing such temporal distributions and are used widely in tasks such as speech recognition (Rabiner, 1990), computational genomics (Eddy, 2004) and decoding neural spike data (Escola et al., 2011). More recently, HMMs have begun making their way into the circadian field, effectively modeling rhythms of both molecular (Bieler et al., 2014) and behavioral data (Harper, 2017). We expect such probabilistic frameworks to be crucial for understanding the relationships between circadian clock components across all levels of the system.

## Discussion

Complexity exists at all levels of the circadian system. Multiple TTFL maintain the ticking of the individual cellular clocks. This timing can then be communicated, neurochemically or electrically, to other cells that use the information to set the phase of their own internal clocks and orchestrate downstream processes. However, the flow of this information is not unidirectional. Multiple central oscillators exchange timing information set by incoming sensory information to compute time. This raises a further question of how the circadian system weights each oscillator’s contribution (a problem well-suited to probabilistic modeling).

Existing methods used to quantify circadian function are imperfect. They are typically measured in free running conditions in order to avoid masking effects that occur as a stimulus response to environmental condition transitions. This necessarily removes the context in which circadian clocks operate and results in rapidly dampened rhythms—both molecular and behavioral. Ideally the phase of the circadian system would be measured during the entrainment period to alleviate the rapid dampening effects of free run, and also reducing the overall length of the experiment. Again, probabilistic models pose an interesting tool for extracting key circadian metrics—such as phase, period and rhythm strength—from noisy time series data in the presence of changing Zeitgebers.

Finally, a better quantitative understanding of the computations—and possible conflicts—occurring in the circadian system is also relevant in the context of the multitude of clock-related pathologies in humans (Roenneberg and Merrow, 2016). These pathologies may be the result of the circadian system simply making the wrong decisions, e.g., due to conflicting data (light at night, social jetlag, trans-meridian travels, etc.). A deeper understanding of where the errors in the decision making processes occur may be a decisive first step toward identifying and treating these pathologies.

## Author Contributions

JS, RH and JA together wrote and designed different aspects of the article.

## Conflict of Interest Statement

The authors declare that the research was conducted in the absence of any commercial or financial relationships that could be construed as a potential conflict of interest.
